# Cortisol Regulates Acid Secretion of H^+^-ATPase-rich Ionocytes in Zebrafish (*Danio rerio*) Embryos

**DOI:** 10.3389/fphys.2015.00328

**Published:** 2015-11-17

**Authors:** Chia-Hao Lin, Tin-Han Shih, Sian-Tai Liu, Hao-Hsuan Hsu, Pung-Pung Hwang

**Affiliations:** ^1^Institute of Cellular and Organismic Biology, Academia SinicaTaipei, Taiwan; ^2^National Institute for Basic Biology, National Institutes of Natural SciencesOkazaki, Japan; ^3^Biodiversity Research Center, Academia SinicaTaipei, Taiwan; ^4^Department of Life Science, National Taiwan Normal UniversityTaipei, Taiwan

**Keywords:** H^+^-ATPase, acid secretion, ionocytes, cortisol, zebrafish

## Abstract

Systemic acid-base regulation is vital for physiological processes in vertebrates. Freshwater (FW) fish live in an inconstant environment, and thus frequently face ambient acid stress. FW fish have to efficiently modulate their acid secretion processes for body fluid acid-base homeostasis during ambient acid challenge; hormonal control plays an important role in such physiological regulation. The hormone cortisol was previously proposed to be associated with acid base regulation in FW fish; however, the underlying mechanism has not been fully described. In the present study, mRNA expression of acid-secreting related transporters and *cyp11b* (encoding an enzyme involved in cortisol synthesis) in zebrafish embryos was stimulated by treatment with acidic FW (AFW, pH 4.0) for 3 d. Exogenous cortisol treatment (20 mg/L, 3 d) resulted in upregulated expression of transporters related to acid secretion and increased acid secretion function at the organism level in zebrafish embryos. Moreover, cortisol treatment also significantly increased the acid secretion capacity of H^+^-ATPase-rich cells (HRCs) at the cellular level. In loss-of-function experiments, microinjection of glucocorticoid receptor (GR) morpholino (MO) suppressed the expression of acid-secreting related transporters, and decreased acid secretion function at both the organism and cellular levels; on the other hand, mineralocorticoid receptor (MR) MO did not induce any effects. Such evidence supports the hypothesized role of cortisol in fish acid-base regulation, and provides new insights into the roles of cortisol; cortisol-GR signaling stimulates zebrafish acid secretion function through transcriptional/translational regulation of the transporters and upregulation of acid secretion capacity in each acid-secreting ionocyte.

## Introduction

Defects in acid-base regulation in mammals are related to certain syndromes, such as bone demineralization, urinary stones, and hypocalcemia (Karet, 2002). As such, vertebrates must maintain the systemic acid-base balance to ensure survival. Freshwater (FW) fish live in an aquatic environment that exhibits fluctuations in ionic compositions and pH levels. Because the buffering capacity of FW is much lower than that of marine water, the pH value of FW is very sensitive to ambient factors, including natural acidification, acid rain, or other pollutants. Ambient acidity results in internal acidosis in fish; as such, fish have to enhance their acid secretion capacity to restore body fluid acid-base homeostasis under such conditions (Evans et al., 2005; Hwang et al., 2011; Guh et al., 2015). In fish, embryonic skin and adult gills are the major organs for acid secretion, and ionocytes are the cells in the skin and gills which carry out the proton transport function (Hwang et al., 2011). In zebrafish, transporters related to acid secretion [H^+^-ATPase, sodium-hydrogen exchanger 3b (NHE3b), anion changer 1b (AE1b), and carbonic anhydrase 2-like a and 15a (CA2-like a and CA15a)] are expressed in H^+^-ATPase-rich cells (HRCs), a specific subtype of ionocyte for acid-base regulation. When translation of any one of these transporters was inhibited in zebrafish embryos, acid secretion was significantly decreased (Horng et al., 2007; Yan et al., 2007; Lin et al., 2008; Lee et al., 2011; Shih et al., 2012). In the proposed model for the acid secretion mechanism in zebrafish HRCs, apical H^+^-ATPase and NHE3b extrude H^+^ from HRCs, the membrane-bound form of CA15a generates H_2_O and CO_2_ from the extruded H^+^ and environmental HCO${}_{3}^{-}$, and the CO_2_ then enters HRCs. The hydration of CO_2_ is catalyzed by cytosolic CA2-like a to form H^+^ and HCO${}_{3}^{-}$, and finally basolateral AE1b transports the cytosolic HCO${}_{3}^{-}$ out of the cell (Hwang and Chou, 2013) Homologs of these transporters have also been found in the gills of other teleost species (Furukawa et al., 2011; Hwang et al., 2011; Hsu et al., 2014).

Metabolic acidosis [caused by HCl infusion (70 mmol L^−1^) or pH4 FW] stimulated acid secretion in the embryos of rainbow fish and zebrafish embryos (Horng et al., 2009; Gilmour et al., 2011). Horng et al. (2009) reported that the HRC density of zebrafish embryos exposed to pH4 FW was increased to enhance acid secretion (Horng et al., 2009). Fish with acidosis exhibited enhanced levels of circulating cortisol (Brown et al., 1986; Wood et al., 1999; Warren et al., 2004; Ivanis et al., 2008a; Gilmour et al., 2011; Kumai et al., 2012). Corticosteroids consist of the cortisol and aldosterone. Teleosts lack aldosterone synthase, but have 11β-hydroxylase (encoded by *cyp11b*) to undertake the final step of cortisol synthesis (Mornet et al., 1989; Bury and Sturm, 2007). Cortisol is a vital hormone for fish osmoregulation because it is their major corticosteroid. Cortisol mediates ion regulation through stimulating ionocyte proliferation and differentiation in FW teleosts (Evans et al., 2005; Cruz et al., 2013a,b). Therefore, increased cortisol levels may increase acid secretion in FW teleosts with acidosis. Additionally, the effect of cortisol on transporters involved in acid secretion has also investigated by several research groups. In trout, cortisol treatment for 2 or 4 d enhanced the mRNA expression of branchial NHE2 and H^+^-ATPase (AL-Fifi, 2006; Ivanis et al., 2008b). The expression of H^+^-ATPase and NHE3b was also stimulated in 1-d post fertilization (dpf) zebrafish embryos by cortisol treatment (Cruz et al., 2013a). In contrast, branchial cytosolic CA expression was not modulated by 48-h cortisol infusion in rainbow trout (Gilmour et al., 2011). Cortisol treatment for 2-d did not regulate NHE3 expression in 4-dpf zebrafish embryos (Kumai et al., 2012). Some studies also addressed the effect of cortisol on the activity of transporters related to acid secretion: chronic cortisol treatment for 7-d was reported to increase the activity of branchial H^+^-ATPase, and cortisol infusion for 48 h stimulated branchial CA activity (Lin and Randall, 1993; Gilmour et al., 2011). As such, although cortisol stimulates ionocyte proliferation, its effect on transporters involved in acid secretion seems to be inconsistent. Acid secretion by teleosts is cooperatively executed by transporters involved in acid secretion that are expressed in ionocytes. Therefore, comprehensive and integral study is required to elucidate the effects of cortisol on such transporters and acid secretion in fish.

Incubation in pH4 FW not only increased the density of HRCs, but also enlarged the apical region of HRCs (Horng et al., 2009). Modification of the apical surfaces of ionocytes in teleosts is related to the ambient ionic composition (Hwang and Lee, 2007). Horng et al. (2009) further provided direct evidence for the nature of the relationship between acid secretion and apical surface of HRCs in zebrafish embryos after exposure to pH4 FW (Horng et al., 2009). Acid FW resulted in an increase of whole-body cortisol in zebrafish embryos, and the apical area of ionocytes was significantly increased by exogenous cortisol treatment in trout and salmon (Laurent and Perry, 1990; Perry et al., 1992). Based on these studies, it appears that cortisol may directly regulate acid secretion at the single ionocyte level, but no direct evidence is currently available. Other studies have shown the actions of corticosteroid hormone on the expression and/or activity of ion transporters. Aldosterone regulates NH_3_ excretion and acid secretion by differentially modulating anion exchanger 1 (AE1), H^+^-ATPase, H^+^-K^+^-ATPase, pendrin, and Rhcg in mammalian intercalated cells (Wagner et al., 2009; Izumi et al., 2011). It has also been shown that the activity and expression of NHE3 in Caco-2 cells are stimulated by dexamethasone, a cortisol analog (Wang et al., 2007). On the contrary, dexamethasone treatment inhibited the activity of purified human CA-I and CA-II (Alım et al., 2015). Taken together, these data raise the possibility that cortisol may directly modulate acid secretion at the single ionocyte level through regulating transporters related to acid secretion in vertebrates; however, this hypothesis remains to be confirmed by convincing *in vivo* evidence. Cortisol was able to upregulate transcription activity of glucocorticoid response element (GRE) containing reporter plasmid in cell lines experiment with transfected teleost GR and MR (Bury et al., 2003; Greenwood et al., 2003; Sturm et al., 2005). Gene knockdown experiments using zebrafish and medaka were performed to show that ion transporter expression and ionic homeostasis in proliferating ionocytes were disturbed by disrupting glucocorticoid receptor (GR), but not mineralcorticoid receptor (MR; Lin et al., 2011; Cruz et al., 2013b; Trayer et al., 2013). GR seems to be a major factor for mediating the effect of cortisol on fish ion regulation. Cortisol stimulates ionocyte proliferation, but its effects on transporters involved in acid secretion in teleosts are diverse (see above). The effects of GR and MR on these transporters also require further exploration. Furthermore, it is still unknown whether GR and MR regulate acid secretion at the single ionocyte level.

Zebrafish is able to survive in FW with pH as low as 4, and transporters related to acid secretion have been well identified in zebrafish ionocytes (Hwang and Chou, 2013). In addition, zebrafish has several advantages, such as the availability of non-invasive electrophysiological, molecular, and bioinformatic approaches (Guh et al., 2015), which make it a competent model with which to study the hormone control of acid secretion in fish. Our understanding of the how cortisol regulates acid secretion of fish is fragmentary until now. Furthermore, it is still unknown whether cortisol and its receptors (GR and MR) can regulate acid secretion at the single ionocyte level. Therefore, we clarified these issues by using zebrafish as a model in the present study. Herein, we aimed to answer the following questions: (1) Does cortisol exert its actions on acid secretion through affecting mRNA expression of transporters (H^+^-ATPase, NHE3b, AE1b, CA2-like a, and CA15a) in zebrafish embryos? (2) Does cortisol regulate these transporters through GR and/or MR? (3) Do cortisol and its receptor directly regulate acid secretion at the single ionocyte level? Addressing these questions promises to provide us an overall and detailed understanding of how cortisol regulates fish body fluid acid-base homeostasis.

## Materials and methods

### Experimental animals

Zebrafish (*Danio rerio*) were kept in local tap water at 28.5°C under a 14:10-h light-dark photoperiod at the Institute of Cellular and Organismic Biology, Academia Sinica, Taipei, Taiwan. Experimental protocols were approved by the Academia Sinica Institutional Animal Care and Utilization Committee (approval no.: RFIZOOHP220782).

### Acclimation experiments

Local tap water (FW, pH 7.0–7.05) and acidic FW (AFW, pH 4.00–4.05) were prepared to determine the effects of an acidic medium. The acidic medium was made by adding H_2_SO_4_ to FW, while the concentrations of other ions in AFW were the same as those in FW. Fertilized zebrafish eggs were transferred to either FW or AFW, and incubated thereafter until sampling at 3 d post-fertilization (dpf). Media were changed twice every day. The pH values and ion concentrations of all experimental media were verified using a pH meter (MP225; Mettler-Toledo, Schwer-zenbach, Switzerland) and an atomic absorption spectrometer (U-2000; Hitachi, Tokyo, Japan), respectively.

### Cortisol incubation experiments

For cortisol incubation experiments, we referred to cortisol dosages used in previous studies (Kiilerich et al., 2007; Lin et al., 2011; Cruz et al., 2013a). Cortisol (hydrocortisone, H4881, Sigma Chemical Co., St Louis, MO, USA) was dissolved in local tap water at 0 (control) and 20 mg/l. Zebrafish embryos were incubated in the cortisol media immediately after fertilization, and were sampled at 3 dpf for the subsequent analysis. The incubation media were changed with new cortisol solution every day to maintain constant levels of cortisol. During incubation, neither significant mortality nor abnormal behavior was observed.

### RNA extraction

After anesthetization with 0.03% MS222, appropriate amounts of zebrafish embryos were sampled and homogenized in 1 ml Trizol reagent (Invitrogen, Carlsbad, CA, USA), mixed with 0.2 ml chloroform, and then thoroughly shaken. Supernatants were obtained by centrifugation at 4°C and 12,000 × g for 30 min. The samples were then mixed with an equal volume of isopropanol. Pellets were precipitated by centrifugation at 4°C and 12,000 × g for 30 min, washed with 70% alcohol, and then stored at −20°C until use.

### Reverse-transcription polymerase chain reaction (RT-PCR) analysis

For complementary (c)DNA synthesis, 1–5 μg of total RNA was reverse-transcribed in a final volume of 20 μl containing 0.5 mM dNTPs, 2.5 μMoligo (dT)_20_, 250 ng random primers, 5 mM dithiothreitol, 40 units RNase inhibitor, and 200 units Superscript RT (Invitrogen) for 1 h at 50°C, followed by incubation at 70°C for 15 min.

### Quantitative real-time PCR (qPCR)

A Light Cycler real-time PCR system (Roche, Penzberg, Germany) was used to perform qPCR in a final reaction volume of 10 μl, containing 5 μl 2x SYBR Green I Master Mix (Roche Applied System), 300 nM of the primer pairs, and 20–30 ng cDNA. The standard curve for each gene was checked in a linear range with β-actin as an internal control. The primer sets for the qPCR followed previous studies (Yan et al., 2007; Lin et al., 2008, 2011; Lee et al., 2011).

### Morpholino oligonucleotide (MO) knockdown and rescue

The zebrafish MR MO (5′-AAC TTTGGTATCTTTTAGTCTCCAT-3′) and GR MO, was designed against the *gr* splicing variant, (5′-CTGCTTCATGTATT TTAGGGTTCCG-3′) were designed as previously described (Lin et al., 2011). A standard control MO (5′-CCTCTTACCTC AGTTACAATTTATA-3′) was used as the control. The GR MO (3.5 ng/embryo) and MR MO (3.5 ng/embryo) were injected into embryos at the 1–2 cell stage using an IM-300 microinjector system (Narishige Scientific Instrument Laboratory, Tokyo, Japan). MO-injected embryos were sampled at 3 dpf for subsequent analyses. Rescue experiments for the defects caused by the GR MO were performed by synthesizing cRNA from a pCS2+ plasmid containing full-length GR. The expression plasmid was linearized and used as template for cRNA synthesis using an SP6 message RNA polymerase kit (Ambion, Austin, TX, USA). Finally, the full-length GR cRNA (300 pg/embryo) and GR were co-injected into embryos at the 1–2 cell stage, and embryos were sampled at 3 dpf.

### Scanning ion-selective electrode technique (SIET)

In the present study, we used the SIET technique to detect H^+^ flux at the surface of zebrafish larvae. The method was performed largely as described previously (Shih et al., 2008, 2012). In brief, micropipettes with tip diameters of 3–4 μm were pulled by a Sutter P-97 Flaming Brown pipette puller (Sutter Instruments, San Rafael, CA). Micropipettes were then baked at 120°C overnight and coated with dimethyl chlorosilane (Sigma-Aldrich) for 30 min. To make an ion-selective microelectrode (probe), micropipettes were backfilled with a 1-cm column of electrolytes and frontloaded with a 20–30-μm column of liquid ion-exchange cocktail (Sigma-Aldrich). The following ionophore cocktail and electrolytes were used: H^+^ ionophore I cocktail B (40 mM KH_2_PO_4_ and 15 mM K_2_HPO_4_; pH 7). To calibrate the ion-selective probe, the Nernstian property of each microelectrode was measured by placing the microelectrode in a series of standard solutions (pH 6, 7, and 8 for the H^+^ probe). By plotting the voltage output of the probe against [H^+^] values, a linear regression yielded a Nernstian slope of 58.6 ± 0.8 (*n* = 10) for H^+^.

### Measurement of surface H^+^ gradients in the skin

SIET was performed at room temperature (26–28°C) in a small plastic recording chamber filled with 1 ml of normal recording medium containing 0.5 mM NaCl, 0.2 mM CaSO_4_, 0.2 mM MgSO_4_, 0.16 mM KH_2_PO_4_, 0.16 mM K_2_HPO_4_, 300 μM MOPS buffer, and 0.3 mg/l ethyl 3-aminobenzoate methanesulfonate (Tricaine, Sigma-Aldrich). The pH of the recording media was adjusted to 7.0 by adding NaOH or HCl solution. Before measurement, an anesthetized larva was positioned in the center of the chamber with its lateral side in contact with the base of the chamber. After 3 min of waiting for signal stabilization, the ion-selective probe was moved to the target position (10–20 μm away from the yolk sac membrane) to record the ionic activities for 10 s; the probe was then immediately moved away (~1 cm) to record the background for another 10 s. The averaged voltage (mV) from the 10 s serial recording was used to calculate the ionic concentration at target or background. To calculate ionic gradients, the background concentration was subtracted from the concentration at the target. In this study, Δ[H^+^] was used to represent the measured H^+^ gradients between the targets (at the surface of larval skin) and background. Lin et al. (2006) showed that the measured ionic gradients vary with the distance between the probe tip and the larval skin (Lin et al., 2006); therefore, the probe's location was maintained as consistently as possible for all samples. The noise of the system was usually < 10 μV and was neglected when calculating the ionic gradients (the recorded voltage difference with larvae was usually 1–10 mV).

### Measurement of H^+^ flux in single ionocytes

To record the H^+^ flux at the surface of HR cells, the microelectrode was moved to a position 2 μm above the surface of a cell. At every position, the voltage difference in microvolts was measured by probing orthogonally to the surface at 10-μm intervals. The recording was performed for 10 replicates, and the median of the repeats was used to calculate the ion flux of the cell. To calculate ionic flux, voltage differences were first converted into a concentration gradient ΔC (μmol·l^−1^·cm^−3^). ΔC was subsequently converted into ionic flux using Fick's law of diffusion in the following equation: *J* = D(ΔC)/ΔX, where *J* (pmol·cm^−2^·s^−1^) is the net flux of the ion, D is the diffusion coefficient of the ion (2.09 × 10^−5^ cm^2^/s for H^+^), and ΔX (cm) is the distance between the two points.

### Statistical analysis

Group data sets were confirmed to be normally distributed by Anderson Darling Normality Test (*p* < 0.05). Data are presented as the mean ± SD and were analyzed by One-way analysis of variance (ANOVA) and Student's *t*-test.

## Results

### Expression of target gene mRNA in zebrafish embryos exposed to acidic FW (AFW)

**Figure 1:**
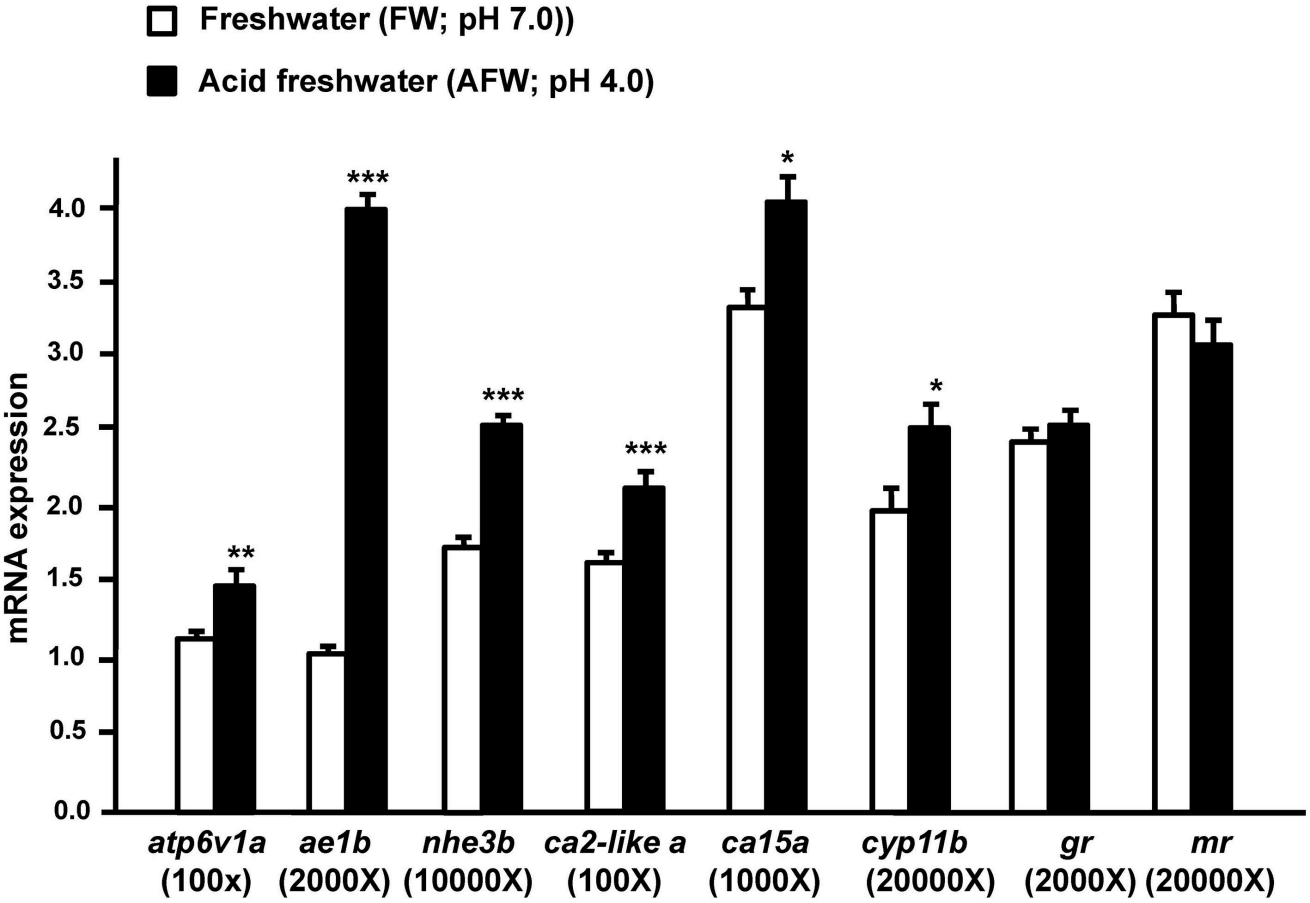
**The effect of FW (pH 7.0) and AFW (pH 4.0) on mRNA expression of the indicated genes in 3d post-fertilization (dpf) zebrafish embryos**. The expression of *atp6v1a, ae1b, nhe3b, ca2-like a, ca15a, cyp11b, gr*, and *mr* mRNA was analyzed by qPCR, and the values were normalized to β-actin. Values are the mean ± SD (*n* = 6). Student's *t*-test, ^*^p < 0.05; ^**^p < 0.01; ^***^p < 0.001.

To explore the effect of acidic water on transporters involved in acid secretion in zebrafish embryos, we exposed 3 dpf zebrafish embryos to FW (pH 7.0) or AFW (pH 4.0). Exposure to AFW resulted in significantly greater expression of *atp6v1a, nhe3b, ae1b, ca2-like a*, and *ca15a*, as compared to expression in fish exposed to FW. On the other hand, *cyp11b*, encoding a cortisol-synthesis enzyme, exhibited enhanced expression in fish exposed to AFW. However, expression of *gr* and *mr* were no different between the FW and AFW groups (Figure 1).

### Zebrafish embryos treated with exogenous cortisol exhibited altered (I) expression of mRNA encoding transporters related to acid secretion and (II) proton secretion

**Figure 2:**
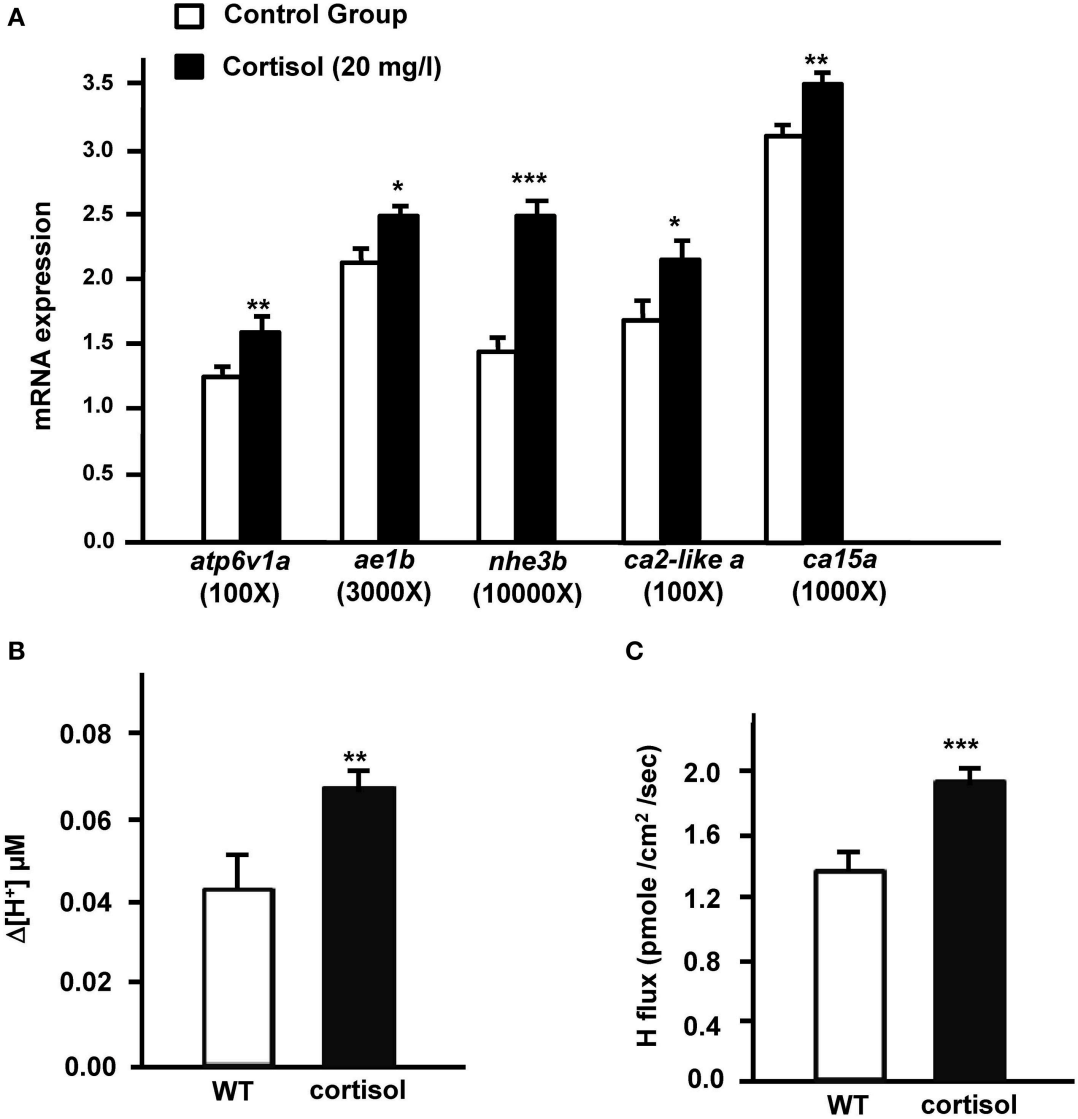
**Effects of exogenous cortisol on (A) the mRNA expression of transporter genes, (B) proton secretion in the embryonic skin, and (C) proton secretion at single H^+^-ATPase-rich cells (HRCs) in 3-dpf zebrafish embryos**. The expression of mRNA was analyzed by qPCR using β-actin as the internal control. SIET was used to measure the H^+^ activity at the skin and at single cells. Values are the mean ± SD (*n* = 6–12). Student's *t*-test, ^*^p < 0.05; ^**^p < 0.01; ^***^p < 0.001.

Expression of *cyp11b* was dramatically upregulated in AFW. Therefore, we investigated the effect of cortisol on (i) transporters related to acid secretion and (ii) proton secretion in zebrafish embryos. In the present study, expression of *atp6v1a, nhe3, ae1b, ca2-like a*, and *ca15a* were evidently stimulated by exogenous cortisol treatment in 3 dpf zebrafish embryos (Figure 2A). The proton secretion of zebrafish embryos was also considerably enhanced by cortisol (Figure 2B). We further explored the effect of cortisol on proton secretion by examining the function of HRCs. As shown in Figure 2C, proton secretion of HRCs was significantly stimulated by exogenous cortisol treatment in zebrafish embryos (Figure 2C).

### Effects of GR or MR knockdown on (I) mRNA expression of transporters related to acid secretion and (II) proton secretion in GR and MR morphant zebrafish embryos

**Figure 3:**
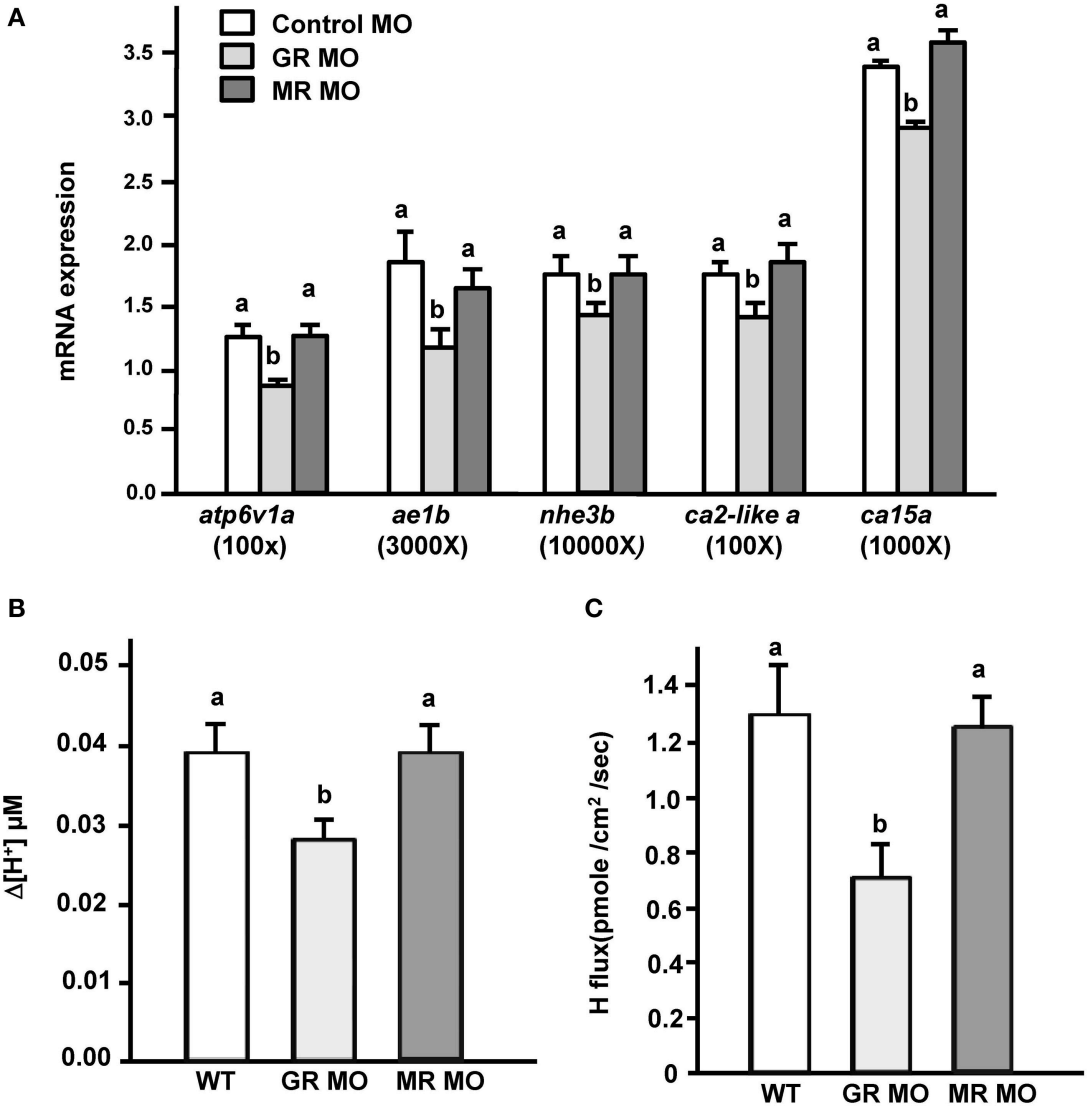
**Effects of mineralocorticoid receptor (MR) and glucocorticoid receptor (GR) morpholino oligonucleotides (MO) on (A) mRNA expression of transporters, (B) proton secretion in the embryonic skin, and (C) proton secretion at single H^+^-ATPase-rich cells (HRCs) in 3-dpf zebrafish embryos**. The expression of mRNA was analyzed by qPCR using β-actin as the internal control. SIET was used to measure H^+^ activity at the skin and at single cells. Different letters indicate a significant difference (*p* < 0.05), as determined using One-way ANOVA followed by Tukey's multiple-comparison test. Values are the mean ± SD (*n* = 6–12).

Exogenous cortisol treatment stimulated (i) the expression of transporters related to acid secretion and (ii) proton secretion in zebrafish embryos. Hence, we further explored the effect of cortisol receptor (GR and MR) on acid secretion in zebrafish embryos. Zebrafish fertilized eggs were injected with either GR or MR MO, and transporter expression and proton secretion were analyzed in 3 dpf embryos. In the present study, expression of *atp6v1a, nhe3b, ae1b, ca2-like a*, and *ca15a* were all significantly downregulated in GR morphants, but unaffected in MR morphants (Figure 3A). Similarly, proton secretion by embryos and HRC was only inhibited in GR morphant zebrafish embryos (Figures 3B,C).

### Effects of GR overexpression on (I) mRNA expression of transporters related to acid secretion and (II) acid secretion in GR morphant zebrafish embryos

**Figure 4:**
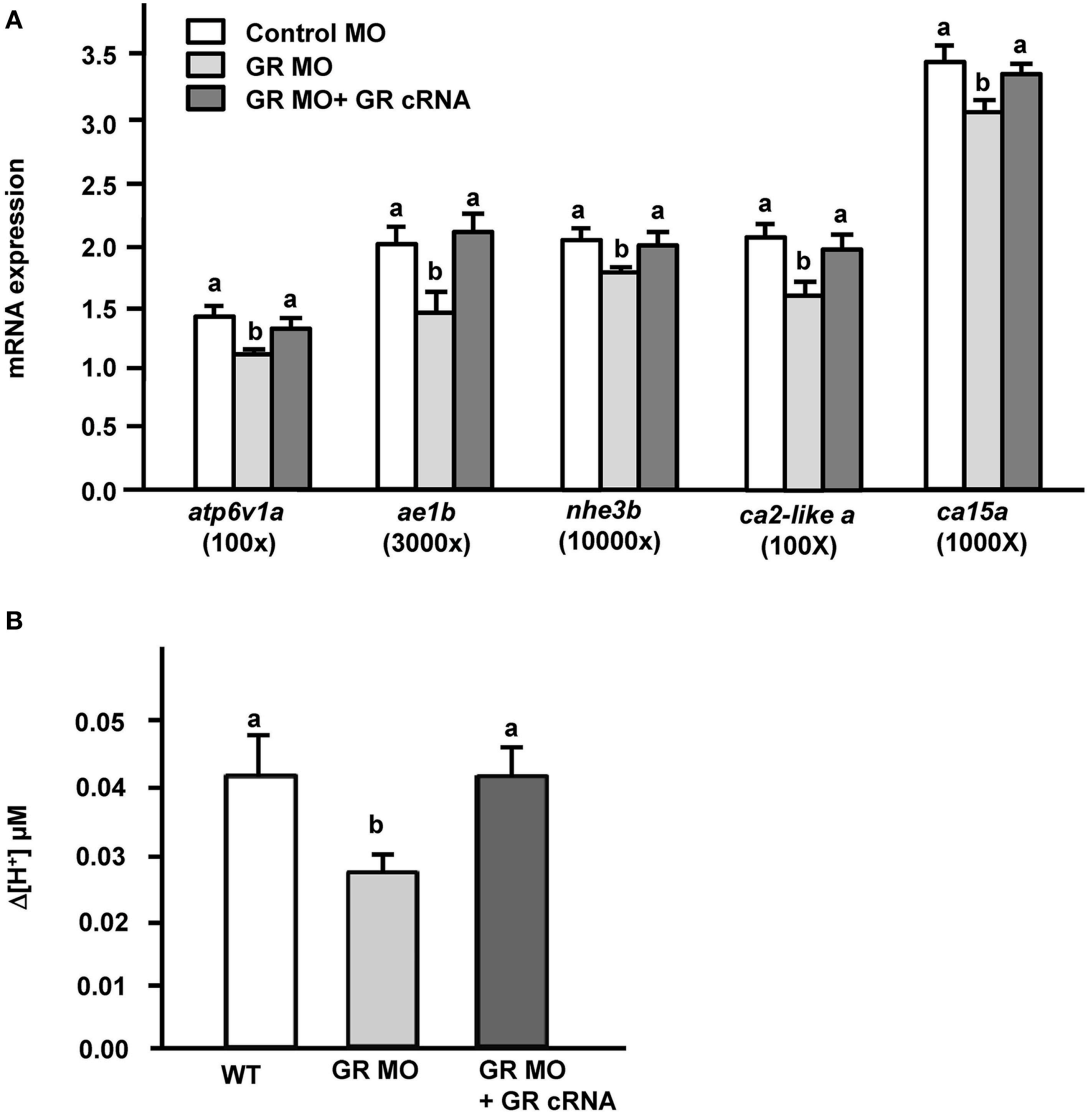
**Effects of GR overexpression on (A) mRNA expression of transporters and (B) proton secretion in the embryonic skin in 3-dpf GR morphant zebrafish embryos**. Expression of mRNA was analyzed by qPCR using β-actin as the internal control. SIET was used to measure H^+^ activity at the skin. Different letters indicate a significant difference (*p* < 0.05), as determined using One-way ANOVA followed by Tukey's multiple-comparison test. Values are the mean ± SD (*n* = 6–12).

Transporter expression and acid secretion were reduced in GR morphant zebrafish embryos. To further demonstrate the effect of GR on proton secretion, exogenous zebrafish GR cRNA was overexpressed in GR morphant zebrafish embryos. We found that GR gain-of-function could completely reverse the decrease in mRNA expression of transporter genes (*atp6v1a, nhe3b, ae1b, ca2-like a*, and *ca15a*) in GR morphants (Figure 4A). Similarly, the proton secretion of embryos could also be fully rescued in morphants by overexpression of GR (Figure 4B).

### Effect of AFW treatment on (I) mRNA expression of transporters related to acid secretion and (II) proton secretion in GR and MR morphant zebrafish embryos

**Figure 5:**
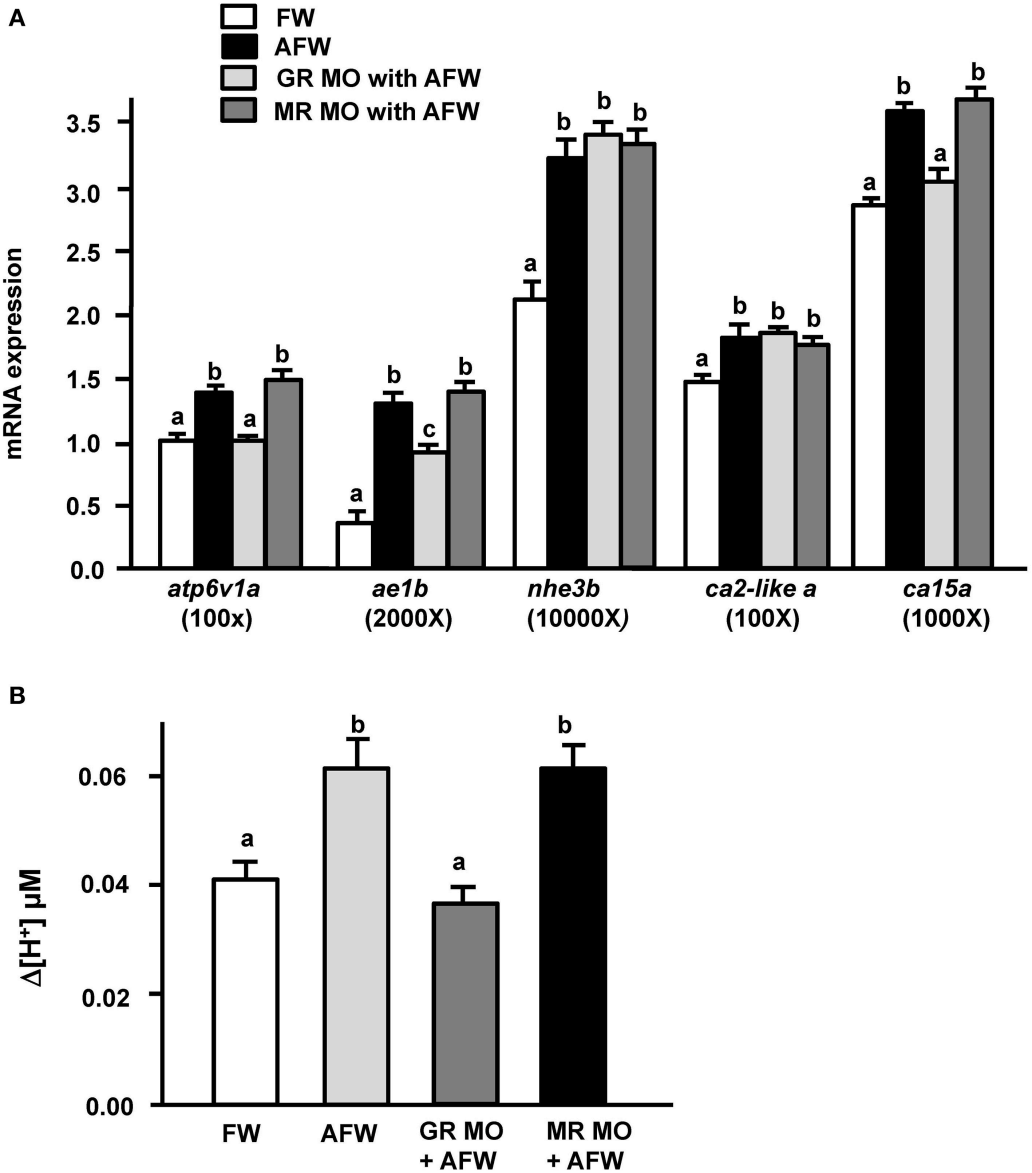
**Effects of MR and GR MOs on (A) mRNA expression of transporters and (B) proton secretion in the embryonic skin in 3-dpf zebrafish embryos treated with FW (pH 7.0) or AFW (pH 4.0)**. Expression of mRNA was analyzed by qPCR using β-actin as the internal control. SIET was used to measure H^+^ activity at the skin. Different letters indicate a significant difference (*p* < 0.05), as determined using One-way ANOVA followed by Tukey's multiple-comparison test. Values are the mean ± SD (*n* = 6–12).

To explore the effect of GR and MR on zebrafish embryos under acid stress, we exposed GR and MR morphant zebrafish embryos to AFW, and then examined (i) the mRNA expression of genes encoding transporters related to acid secretion and (ii) proton secretion in 3 dpf embryos. Consistent with the above results, both the mRNA expression of examined transporters related to acid secretion and proton secretion activity were strongly increased in control MO morphant zebrafish embryos subjected to AFW treatment (Figure 5A). In addition, transporter expression and proton secretion of MR morphant zebrafish were stimulated by AFW (Figure 5A). However, the situation was different in GR morphant embryos treated with AFW. Although *nhe3b* and *ca2-like a* still exhibited enhanced expression, levels of *atp6v1a, ae1b*, and *ca15* mRNA and acid secretion were either not or only partially upregulated by AFW in GR morphant zebrafish embryos (Figure 5B).

## Discussion

In adult zebrafish, AFW treatment enhanced HRC proliferation and the expression of *atp6v1a, ae1b*, and *ca15a*, but did not affect expression of *ca2-like a*, and actually inhibited expression of *nhe3b* in the gills (Yan et al., 2007; Lin et al., 2008; Chang et al., 2009; Horng et al., 2009; Lee et al., 2011). In trout, the mRNA expression of NHE3 and cytosolic CA (an ortholog of zebrafish CA2) showed diverse responses to acidosis in the kidney and gills (Ivanis et al., 2008a,b; Gilmour et al., 2011). In zebrafish embryos, acid secretion and HRC proliferation are stimulated by 4-d AFW treatment (Chang et al., 2009; Horng et al., 2009). The enhanced acid secretion in zebrafish subjected to acidic challenge resulted from upregulation of the mRNA expression of acid-secreting transporters and enzymes. In support of this notion, mRNA expression of all genes identified so far (*apt6v1a, nhe3b, ae1b, ca2-like a*, and *ca15a*; Figure 1) and acid secretion activity were simultaneously and significantly stimulated by AFW in zebrafish embryos (Chang et al., 2009; Horng et al., 2009). However, the responses of *nhe3b* and *ca2-likea* appear to differ between the embryos and adult gills. This difference may be because zebrafish embryos express *nhe3b* and *ca2-like a* not only in the skin and gills, but also in other tissues, such as kidney, and whole embryos are used for RT-PCR analyses (Yan et al., 2007; Lin et al., 2008).

Cortisol levels were previously reported to be upregulated in zebrafish embryos treated with AFW (Kumai et al., 2012). The mutation of *cyp11b*, encoding the enzyme for final step of cortisol synthesis, caused cortisol deficiency in the patients with congenital adrenal hyperplasia (Krone et al., 2005). In the present study, we observed that the expression of *cyp11b*, was also stimulated by AFW. Therefore, these findings suggest that AFW treatment stimulates *cyp11b* transcription, thereby enhancing cortisol synthesis in zebrafish. In addition, the present study further demonstrates that cortisol treatment can stimulate acid secretion function in zebrafish embryos. Taken together, the data indicate that the increase in cortisol level upon AFW treatment stimulates the acid secretion ability of zebrafish. Transporters related to acid secretion that are expressed in the gill ionocytes are vital for the acid secretion mechanism in fish. This is the first integrative study to demonstrate that cortisol treatment resulted in significant upregulation of mRNA encoding the related transporter and enzymes, H^+^-ATPase, NHE3b, CA2-like a, CA15 and AE1b, in zebrafish embryos (Figure 2A). The mRNA expression or activity of renal or branchial H^+^-ATPase and NHE2/3 was also stimulated by cortisol treatment in trout (Lin and Randall, 1993; AL-Fifi, 2006; Ivanis et al., 2008a,b). On the other hand, information on the effect of cortisol on transporters other than H^+^-ATPase and NHEs was previously limited or inconsistent. In both zebrafish and medaka, AE1 is specifically expressed in acid-secreting ionocytes, and loss-of-function experiments have indicated that differentiation of these ionocytes is regulated by cortisol-GR signaling (Lee et al., 2011; Cruz et al., 2013a,b; Trayer et al., 2013; Hsu et al., 2014). Direct evidence for the effect of cortisol on AE1 expression has only been obtained for zebrafish. CA15, a membrane-bound CA specifically expressed in the gill/skin acid-secreting ionocytes, has only been identified and functionally analyzed in zebrafish (Lin et al., 2008). The effect of cortisol on membrane-bound CA, a key enzyme for the acid-base regulation mechanism in fish gills (Gilmour, 2012; Guh et al., 2015) is still unclear in fish, but cortisol treatment was reported to stimulate the mRNA expression of membrane-bound CA4 in the kidney of trout (Gilmour et al., 2011). Herein, cortisol treatment was demonstrated to stimulate the mRNA expression of gill/skin ionocyte-specific membrane-bound CA15a in zebrafish. Cytosolic CA2-like a is universally expressed in multiple tissues in zebrafish, but is specifically and dominantly localized to HRCs (Lin et al., 2008). In the present study, the mRNA expression of ionocyte-specific cytosolic CA2-like a was found to be enhanced by cortisol treatment. On the other hand, cortisol treatment affected the activity of cytosolic CA in the gills, but not its mRNA expression in either the gills or kidney of trout (Gilmour et al., 2011). Cytosolic CA2 is responsible for the majority (~95%) of CA activity, while the other membrane-bound CA isoforms support the remaining activity in mammalian kidney (Wistrand and Knuuttila, 1989; Purkerson and Schwartz, 2007). Cortisol appears to stimulate the expression and/or activity of CAs in different ways between trout and zebrafish, but the detailed mechanisms underlying this difference are yet to be clarified.

The molecular and cellular mechanisms of functional regulation of related ion transporters and ionocytes have been studied in detail, particularly under acclimation to acidic environments: acid acclimation induces a compensatory enhancement in acid secretion capacity, which is achieved not only by increasing the acid-secreting function of individual HR cells, but also by increasing HR cell number (Horng et al., 2009); the additional HR cells in acidic FW originate from Gcm2-mediated differentiation of both ionocyte precursor cells and newly proliferating epithelial stem cells (Chang et al., 2009; Horng et al., 2009). Cortisol seems to regulate both pathways to enhance acid secretion function in zebrafish exposed to an acidic environment. Exogenous cortisol treatment was demonstrated to stimulate the differentiation of HRCs with a concomitant increase in acid secretion in the skin of zebrafish embryos (Cruz et al., 2013a,b). The present study further indicated that cortisol directly regulates acid secretion at single HRCs, as surmised through SIET analyses (Figure 2C). To reinforce this effect on acid secretion, cortisol also stimulated the mRNA expression of *atp6v1a, nhe3b, ae1b, ca2-like a*, and *ca15a* in zebrafish embryos (Figure 2A). Several studies also indicated the effects of corticosteroid hormones on the regulation of ion transporter expression and activity. Dexamethasone treatment stimulated NHE3 activity through elevating the mRNA expression of NHE3 and its regulator, SGK1, in human epithelial colorectal adenocarcinoma cells (Wang et al., 2007). Furthermore, the expression of ENaCα is directly regulated by glucocorticoid inhuman kidney and lung (Sayegh et al., 1999). Aldosterone regulates the acid-base balance by differentially modulating H^+^, K^+^-ATPase, H^+^-ATPase, AE1, and Rhcg in mammalian intercalated cells (Izumi et al., 2011). In zebrafish, GR expression was identified in HRCs (Cruz et al., 2013b). Cortisol was previously shown to increase the transcriptional activity of a reporter plasmid co-transfected with teleost GR or MR into a cell line (Trapp and Holsboer, 1996; Colombe et al., 2000; Bury et al., 2003; Greenwood et al., 2003; Sturm et al., 2005). All of these findings suggest that cortisol enhances acid secretion of single HRCs through directly stimulating the expression of transporters related to acid secretion in HRCs. Horng et al. (2009) showed that acid secretion and apical size of HRCs are enhanced by AFW treatment in zebrafish embryos (Horng et al., 2009). Apparently, cortisol is involved in these regulatory events.

Cortisol treatment can induce the transcriptional activity of reporter plasmids in cell lines transfected with teleost GR or MR (Bury et al., 2003; Greenwood et al., 2003; Sturm et al., 2005). Cortisol was suggested to execute its physiological function through GR and/or MR. In the present study, *atp6v1a, nhe3b, ae1b, ca2-like a*, and *ca15a* expression and acid secretion (at the skin and the single cell levels) were not affected in MR morphant zebrafish embryos (Figure 3), or in MR morphants treated with AFW (Figure 5). These results are consistent with previous suggestions that MR plays no or a minor role in fish ionregulation (Lin et al., 2011; Kumai et al., 2012; Cruz et al., 2013b; Takahashi and Sakamoto, 2013; Trayer et al., 2013). Cruz et al. (2013b) indicated that the HRC density of zebrafish embryos was decreased in GR, but not in MR morphants (Cruz et al., 2013b). In the present study, expression of *atp6v1a, nhe3b, ae1b, ca2-like a*, and *ca15a* mRNA were found to be downregulated in GR morphant zebrafish embryos (Figures 3A, 4A); moreover, the acid secretion of whole embryos was also impaired in GR morphants (Figures 3B, 4B). Overexpression (through injection of GR cRNA) rescued the defects observed in GR morphants (Figure 4). These results are consistent with those in the study by Cruz et al. (2013b). Here, we observed that acid secretion at single HRCs was also inhibited in GR morphant zebrafish (Figure 3C). As such, cortisol may, directly stimulate acid secretion and the expression of transporters related to acid secretion in zebrafish HRCs via GR, but not MR. It was noted that cortisol induced about 60% increase in proton secretion from the body surface but the increase was only 43% from HRC (Figures 2B,C). This discrepancy may reflect the influence of epidermal keratinocytes. Keratinocyte is the most dominant cell type in body surface of fish. Several studies reported acid secretions in keratinocytes of zebrafish and medaka embryos (Wu et al., 2010; Liu et al., 2013; Furukawa et al., 2015). Although the ability of acid secretion in a single keratinocyteis far lower than that in a single HRC, the dominant keratinocytes still play a substantial role in acid secretion of the whole embryos. In addition, Cruz et al. (2013b) indicated that the density of epidermal keratinocytes was inhibited in zebrafish GR morphants but without changes in MR morphants. Hence, in the present study we found GR MO showing differential effects on the acid secretions at the organism and the cellular levels (Figures 3B,C).

In the present study, AFW stimulated the mRNA expression of *nhe3b* and *ca2-like a*, even in GR morphant zebrafish embryos (Figure 5A). In addition to cortisol, isotocin and stanniocalcin-1 (STC-1) have also been reported to affect acid secretion function and HRC proliferation in zebrafish (Chou et al., 2011, 2015; Cruz et al., 2013a). Mutual regulation between calciotropic hormones, cortisol, STC-1, and parathyroid hormone 1 was reported in zebrafish embryos (Lin et al., 2011, 2014; Kumai et al., 2015). The counterbalance among cortisol, isotocin, STC-1, and/or other hormones may affect the phenotypes (in terms of acid secretion mechanism) of GR zebrafish morphants treated with AFW. These hormones may mutually cooperate with cortisol to differently regulate the expression of acid-secreting related transporters under acidic water. In GR morphants, a new balance among hormones may be reestablished. Finally, this new balance resulted in upregulated expressions of *nhe3b, ae1b* and *ca2-like a* expression, but decreased expressions of *atp6v1a* and *ca15a*. A subtle change in hormone balance may differentially modulate transporters related to acid secretion to maintain body fluid acid-base homeostasis. Needless to say, more studies will be required to clarify this issue in the future.

In summary, the synthesis and/or release of cortisol in zebrafish is stimulated under AFW challenge, and the increased cortisol level may act via GR to enhance HRC proliferation and the expression of transporters related to acid secretion in HRCs, thereby increasing the acid secretion capacity of the whole organism to overcome internal acidosis. The present study provides new insights into the molecular/cellular mechanisms of cortisol's action on fish body fluid acid-base homeostasis.

### Conflict of interest statement

The authors declare that the research was conducted in the absence of any commercial or financial relationships that could be construed as a potential conflict of interest.
